# Generalized Stacking Fault Energy of Al-Doped CrMnFeCoNi High-Entropy Alloy

**DOI:** 10.3390/nano10010059

**Published:** 2019-12-26

**Authors:** Xun Sun, Hualei Zhang, Wei Li, Xiangdong Ding, Yunzhi Wang, Levente Vitos

**Affiliations:** 1 Applied Materials Physics, Department of Materials Science and Engineering, Royal Institute of Technology, SE-100 44 Stockholm, Sweden; xunsun@kth.se (X.S.); wei2@kth.se (W.L.); levente@kth.se (L.V.); 2 Frontier Institute of Science and Technology, State Key Laboratory for Mechanical Behavior of Materials, Xi’an Jiaotong University, Xi’an 710049, China; dingxd@mail.xjtu.edu.cn; 3 Department of Materials Science and Engineering, The Ohio State University, 2041 College Road, Columbus, OH 43210, USA; wang.363@osu.edu; 4 Division of Materials Theory, Department of Physics and Materials Science, Uppsala University, P.O. Box 516, SE-75120 Uppsala, Sweden; 5 Research Institute for Solid State Physics and Optics, Wigner Research Center for Physics, P.O. Box 49, H-1525 Budapest, Hungary

**Keywords:** high-entropy alloys, generalized stacking fault energy, first-principles, interfacial energy

## Abstract

Using first-principles methods, we investigate the effect of Al on the generalized stacking fault energy of face-centered cubic (fcc) CrMnFeCoNi high-entropy alloy as a function of temperature. Upon Al addition or temperature increase, the intrinsic and extrinsic stacking fault energies increase, whereas the unstable stacking fault and unstable twinning fault energies decrease monotonously. The thermodynamic expression for the intrinsic stacking fault energy in combination with the theoretical Gibbs energy difference between the hexagonal close packed (hcp) and fcc lattices allows one to determine the so-called hcp-fcc interfacial energy. The results show that the interfacial energy is small and only weakly dependent on temperature and Al content. Two parameters are adopted to measure the nano-twinning ability of the present high-entropy alloys (HEAs). Both measures indicate that the twinability decreases with increasing temperature or Al content. The present study provides systematic theoretical plasticity parameters for modeling and designing high entropy alloys with specific mechanical properties.

## 1. Introduction

High-entropy alloys (HEAs) have attracted significant attention in recent years [1,2,3,4,5,6,7]. Excellent combination of strength-ductility properties is one of the great advantages of the face-cubic centered (fcc) HEAs [1,2], which is usually attributed to the deformation twins [1,8].

In conventional alloys, it is often a challenge to improve the strength and ductility at the same time. Usually higher strength is achieved by sacrificing ductility and vice versa. On the other hand, the deformation twinning mechanism can be used to overcome the strength-ductility trade-off. The deformation twins are created by the dislocation gliding in the slip systems under external stress. The newly created twin boundaries hinder the dislocation motion, resulting in an increased work hardening rate (“dynamic Hall-Petch effect”). At the same time, twinning maintains the elongation of alloys during work hardening by delaying the onset of plastic instability by necking [9].

The generalized stacking fault energy (GSFE) plays an important role in understanding the deformation mechanism of fcc alloys [10,11,12]. There are four important parameters of GSFE corresponding to the first four extrema on the energy versus slip vector curve: the unstable stacking fault energy (*γ*_usf_), the intrinsic stacking fault energy (*γ*_isf_), the unstable twin fault energy (*γ*_utf_), and the extrinsic stacking fault energy (*γ*_esf_). *γ*_isf_ is the most widely used parameter to predict the twinning ability, twinning stress [10], and phase stability [13]. Classical theories generally predict that the critical twinning stress ($\tau_{crit}$) is proportional to the intrinsic stacking fault energy *γ*_isf_ [10], i.e., $\tau_{crit}\cdot b_{112}\sim \gamma_{isf}$, where *b*_112_ is the Burgers vector of partial dislocation. A lower *γ*_isf_ suggests that deformation twins are easier to form, and the twinning stress is relatively low. For example, the twinning easily happens in Cu but not in Al, which is attributed to the lower *γ*_isf_ of Cu (45 mJ/m^2^) than that of Al (122 mJ/m^2^) [14]. The measured *γ*_isf_ of many medium-entropy alloys (MEAs) and HEAs are as low as (or even lower than) those obtained for the twinning-induced plasticity (TWIP) steels [10], which is consistent with the large amount of nano-twins observed in these systems. *γ*_isf_ of CrMnFeCoNi HEA is 25–35 mJ/m^2^ [9] and 22–31 mJ/m^2^ [15] measured at room temperature by different works. The deformation twins are easily found at cryogenic temperature in CrMnFeCoNi HEA [1], indicating that *γ*_isf_ decreases with lowering temperature. *γ*_isf_ of MEAs and HEAs were studied by theoretical methods as well. Both CrCoNi and CrMnFeCoNi have negative theoretical *γ*_isf_ [16,17,18], which is ascribed to the metastable character of these alloys [19].

## 2. Methodology

The ab initio calculations were performed using the exact muffin-tin orbitals (EMTO) method [20]. The Perdew–Burke–Ernzerhof (PBE) [21] exchange-correlation functional was adopted to perform the self-consistent and total energy calculations. The chemical and magnetic disorders were treated within the coherent-potential approximation (CPA) [22]. The paramagnetic (PM) state of Al*_y_*(CrMnFeCoNi)_100−*y*_ was modeled within the disordered local magnetic moment approach [23]. The EMTO-CPA method successfully described the lattice constants [24] and the elastic moduli [5] of Al-doped CrMnFeCoNi HEAs in our previous works.

**Figure 1:**
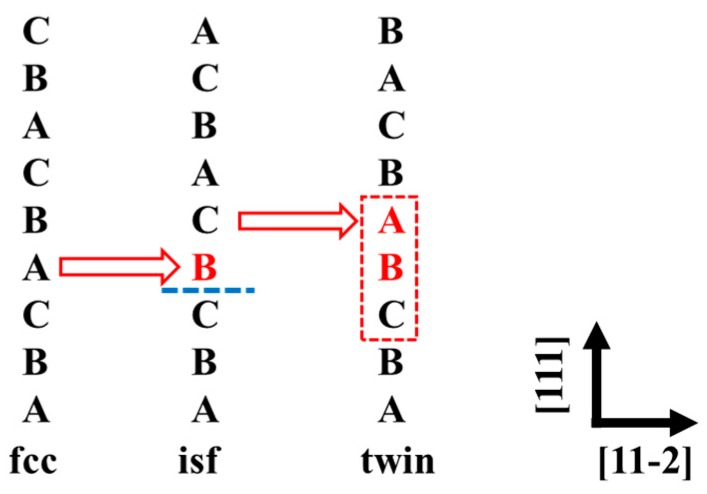
Schematic of planar fault path on {111} planes. Perfect face-cubic centered (fcc) bulk forms an intrinsic stacking fault by shearing one *b*_p_ along <112> direction. Extrinsic stacking fault (twin) formed by shearing one *b*_p_ along <112> direction in the adjacent layer of intrinsic stacking fault.

According to the Mahajan–Chin model [25], the nucleation and propagation of deformation twins in fcc systems by shearing successive {111} planes along the <112> direction [26], as shown in Figure 1. The GSFE was calculated by adopting a 9-layers supercells with and without one fault per unit cell [26]. Due to the periodic boundary condition used, the number of atomic layers needs to be large enough to prevent the influence of the interaction between the two adjacent stacking faults. The 9-layers supercell is proved to be accurate enough for the GSFEs [18]. This approach has been successfully applied in pure metals, binary alloys, and HEAs in previous studies [27,28]. The GSFE was calculated as *γ*_GSFE_ = (*F*^fault^ − *F*^0^)/*A*, where *F*^fault^ and *F*^0^ are the free energies of supercell with and without the fault, respectively, and *A* is the area. The free energy is approximated as *F* = *E* − *TS*_mag_, where *E* is the total energy and *T* is the temperature. Within the mean-field approximation, the magnetic entropy is $S_{mag}\;=\;k_{B}\sum_{i=1}^{6}c_{i}\ln(1+\mu_{i})$, where *k*_B_ is the Boltzmann constant, *c_i_* is the concentration, and *μ_i_* the local magnetic moment of the *i*th alloying element, respectively. The total energy *E* at each temperature and Al concentration was calculated at the corresponding lattice constant. We started from the experimental lattice constants of fcc Al*_y_*(CrMnFeCoNi)_100−*y*_ (*y* = 0, 2, 4, 6, 8) alloys at room temperature [29]. Then we used the coefficient of thermal expansion [30] of fcc CrMnFeCoNi alloy to evaluate the lattice constants of Al*_y_*(CrMnFeCoNi)_100−*y*_ alloys as a function of temperature, i.e., we assumed that Al addition has a negligible influence on the thermal expansion coefficient. This assumption is supported by the fact that the Debye temperature of Al-doped fcc CrMnFeCoNi varies little with the amount of Al [5]. Namely, the Debye temperature changes from 525 to 490 K [5] as the Al concentration increases from 0% to 8%. According to the quasi-harmonic Debye model [31], the corresponding change in the thermal expansion coefficient is less than 10% at 300 K, which leads to less than 0.05% uncertainty in the room-temperature lattice parameters.

## 3. Results and Discussion

**Figure 2:**
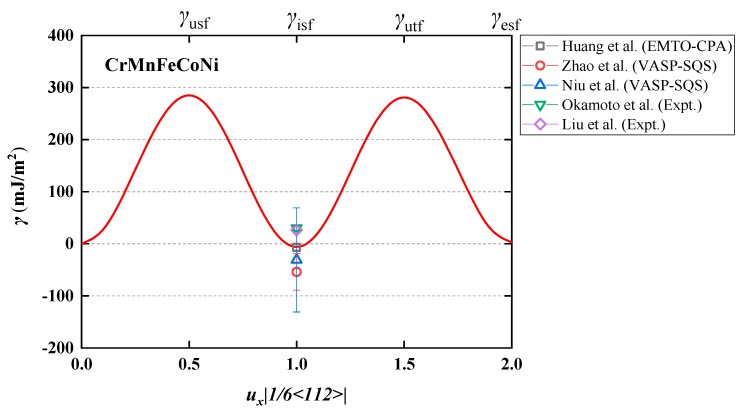
Theoretical generalized stacking fault energy (γ, in mJ/m^2^) of paramagnetic (PM) fcc CrMnFeCoNi alloy calculated at room temperature. The theoretical (exact muffin-tin orbitals-coherent-potential approximation (EMTO-CPA), Vienna ab initio simulation package-special quasi-random structure (VASP-SQS)) [18,33,34] and experimental (Expt.) [9,15] *γ*_isf_ are plotted for comparison.

In Figure 2, we present the GSFE of paramagnetic fcc CrMnFeCoNi at room temperature. For *γ*_usf_, *γ*_isf_, *γ*_utf_, and *γ*_esf_ our predictions give 285, −6, 281, and 3 mJ/m^2^, respectively. We observe that *γ*_isf_ is negative, and *γ*_esf_ is also very small. On the other hand, the energy barriers *γ*_usf_ and *γ*_utf_ are relatively large as compared to the energy barriers obtained for pure metals with low *γ*_isf_ [27], such as Cu, Au, and Ag. We find that the present results of GSFE satisfy with a good accuracy the universal scaling law [32], i.e., $\gamma_{usf}≃\gamma_{utf}-\frac{1}{2}\gamma_{isf}$. It is interesting that although *γ*_isf_ is negative, the universal scaling law remains valid, which suggests that our results are reasonable. Similar negative *γ*_isf_ was reported in previous works. For instance, Huang et al. [18] used a similar approach as the one adopted here and obtained −7 mJ/m^2^ for *γ*_isf_ of PM fcc CrMnFeCoNi at room temperature. On the other hand, the present *γ*_isf_ at 300 K is smaller than the former EMTO result (21 mJ/m^2^) reported by Huang et al. [28]. The relatively large deviation between the two sets of data should be ascribed to the fact that Huang et al. [28] employed the local density appropriation (LDA) instead of the PBE functional adopted here and used an experimental lattice parameter of 3.6 Å compared to 3.59 Å [29] considered here. Furthermore, here we neglect the positive strain contribution to *γ*_isf_, which was considered by Huang et al. [28].

At 0 K and for the ferromagnetic (collinear) state, calculations based on the Vienna ab initio simulation package (VASP) combined with the special quasi-random structure (SQS) approach for the intrinsic stacking fault energy of CrMnFeCoNi gave average values of −54 [33] and −31 mJ/m^2^ [34] with scatter of about ±35 and ±100 mJ/m^2^, respectively. The deviations between the present result and the above VASP values [33,34] can be attributed to the different magnetic states and different alloy theories adopted in those calculations.

**Figure 3:**
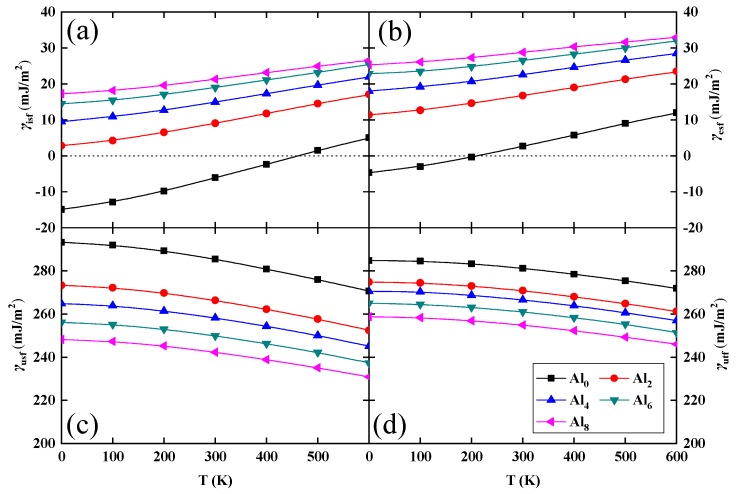
Theoretical generalized stacking fault energy (γ, in mJ/m^2^) of PM fcc Al*_y_*(CrMnFeCoNi)_100−*y*_ (*y* = 0, 2, 4, 6, 8) alloys as a function of temperature and composition. (**a**) The intrinsic stacking fault energy (*γ*_isf_, in mJ/m^2^); (**b**) The extrinsic stacking fault energy (*γ*_esf_, in mJ/m^2^); (**c**) The unstable stacking fault energy (*γ*_usf_, in mJ/m^2^); (**d**) The unstable twinning fault energy (*γ*_utf_, in mJ/m^2^).

In Figure 3, we present the calculated GSFE for the PM fcc Al*_y_*(CrMnFeCoNi)_100−*y*_ (*y* = 0, 2, 4, 6, 8) alloys as a function of temperature and composition. In the considered temperature and Al concentration range, both *γ*_isf_ and *γ*_esf_ increase monotonously with increasing temperature and Al content. At the same time, *γ*_usf_ and *γ*_utf_ decrease with increasing temperature and Al addition. It is found that temperature has a large effect on the GSFE. The values of *γ*_isf_ and *γ*_esf_ for the Al-free alloy are negative when the temperature is below 400 and 200 K, respectively, as shown in Figure 3. We notice that the present temperature dependence of *γ*_isf_ follows closely the one predicted by Huang et al. [28] in spite of the methodological differences discussed above.

**Figure 4:**
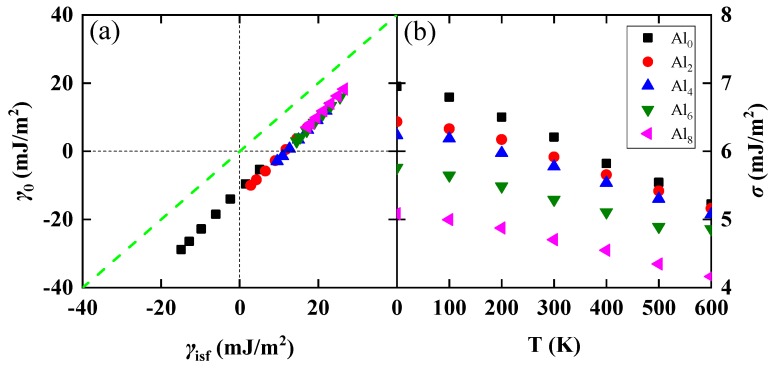
(**a**) Comparison between $\gamma_{isf}$ and $\gamma_{0}$ (in mJ/m^2^) of PM fcc Al*_y_*(CrMnFeCoNi)_100−*y*_ (*y* = 0, 2, 4, 6, 8) alloys. Symbols correspond to temperatures between 0 and 600 K (from left to right) with increments of 100 K. (**b**) The interfacial energy *σ* (in mJ/m^2^) is plotted as a function of temperature and composition.

For elemental metals and homogeneous solid solutions, the intrinsic stacking fault energy can be approximated by the energy difference between the hexagonal close packed (hcp) and fcc lattices, viz. $\gamma_{isf}=\frac{2\left(F^{hcp}-F^{fcc}\right)}{A}+2\sigma$ [13,35], where *F*_hcp_ and *F*_fcc_ are the free energies per atom for the hcp and fcc phase, respectively. The last term is the interfacial contribution describing the transition zone between the fcc matrix and the hcp embryo. The interfacial energy *σ* was estimated to be of the order of 7 mJ/m^2^ [36] for the present Al-free CrMnFeCoNi HEA. In Figure 4a, we compared *γ*_isf_ and the stacking fault energy *γ*_0_ obtained merely from the structural energy difference, viz. $\gamma_{0}=\frac{2\left(F^{hcp}-F^{fcc}\right)}{A}$. We find *γ*_0_ is very close to *γ*_isf_ for all Al concentrations and temperature. Thus, the free energy difference between hcp and fcc can reflect the value of *γ*_isf_. The small difference between *γ*_isf_ and *γ*_0_ is equal to the double of the interfacial energy *σ*. In Figure 4b, we also plotted the interfacial energy $\sigma =(\gamma_{isf}-\gamma_{0})/2$ as a function of temperature. We find that *σ* slightly decreases with increasing temperature and Al content, but it remains in the range of 4–7 mJ/m^2^. Thus, at least for the present alloy family, the composition and temperature dependence of *σ* is rather weak and could safely be omitted. Similar observation was made by Dong et al. using calculations based on floating spin and longitudinal spin fluctuations schemes [37].

Previous theoretical study on the PM CrMnFeCoNi system [38] found that the hcp structure is more stable than the fcc structure below 370 K, which means that the negative values of *γ*_isf_ and *γ*_esf_ shown in Figure 3 are reasonable. Similar to our findings, first-principles calculations [19] discovered that the negative *γ*_isf_ in fcc CrCoNi and CrFeCoNi alloys originate from the thermodynamic stability of the hcp phase at low temperatures. Recently, Zhang et al. [39] confirmed that the hcp phase is more stable thermodynamically than the fcc one at relatively lower temperatures, agreeing well with the theoretical results [38].

**Figure 5:**
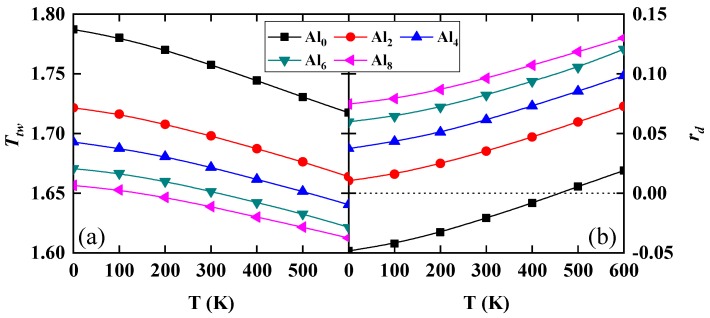
The effect of Al on the twinning ability of PM fcc Al*_y_*(CrMnFeCoNi)_100−*y*_ (*y* = 0, 2, 4, 6, 8) alloys as a function of temperature and composition. Shown are (**a**) *T*_tw_ and (**b**) *r*_d_ as a function of temperature and composition.

Despite the fact that low *γ*_isf_ generally improves the twinning ability of alloys, the combined effects of all energy parameters determining the GSFE should be considered when studying the twinning affinity. That is because the intrinsic energy parameters in Figure 3 exhibit complex temperature and alloying trends. To describe the twinning ability in the Al*_y_*CrMnFeCoNi system, we adopt two twinning ability parameters. The parameter *T*_tw_ proposed by Asaro et al. [40] is defined as

(1) $$T_{tw}=\sqrt{\left(3\gamma_{usf}-2\gamma_{isf}\right)/\gamma_{utf}}$$

when *T_tw_* > 1 a twin is more favorable than the dislocation slip and vice versa. We plot *T_tw_* as a function of temperature and Al content in Figure 5a. We find that all alloys considered here have good twinning ability within the entire temperature range. All *T_tw_* decrease with increasing temperature and Al content, suggesting that the Al addition and increasing temperature decrease the ability of twinning. However, the twinning parameter remains far above 1, meaning that the present alloys remain prone to twinning even when the Al level comes close to the solubility limit within the fcc phase and temperature increases up to 600 K. A second twinning parameter was introduced by Jo et al. [41] as

(2) $$r_{d}=\gamma_{isf}/\left(\gamma_{usf}-\gamma_{isf}\right)$$

In terms of this parameter, one can distinguish four regimes corresponding to different deformation mechanisms. Namely, for *r*_d_ < −0.5 we have stacking fault only, for −0.5 < *r*_d_ < 0 both stacking fault and full slip can be realized, for 0 < *r*_d_ < 2 full slip is combined with twinning, and for *r*_d_ > 2 we have full slip only. Within the range of 0 < *r*_d_ < 2, *r*_d_ = 0 corresponds to the maximum twinning ability. As shown in Figure 5b, all *r*_d_ values are much lower than 2, indicating a strong twinning ability. We find that *r*_d_ increases with increasing Al content and temperature, meaning that the twinning ability decreases, which is fully consistent with the prediction from *T*_tw_. There is one exceptional case that the negative *r*_d_ in Al-free CrMnFeCoNi HEA below 400 K predict that only stacking fault and full slip will happen. However, plenty of deformation twins are found in the CrMnFeCoNi HEA [1,42]. The current twinning parameter cannot explain this phenomenon. Further theory is needed to resolve this question. Byun formula would suggest that the critical stress for twinning also increases and thus it is unclear whether twinning can indeed be realized at elevated temperatures. That depends crucially on the alloy preparation, micro-structure, and strain rate. Describing these effects is a very complex problem and calls for further advanced models built among others on the presently disclosed intrinsic energy parameters.

## 4. Conclusions

Using first-principles alloy theory formulated within the EMTO method, we have calculated the GSFE of paramagnetic fcc Al*_y_*(CrMnFeCoNi)_100−*y*_ (*y* = 0, 2, 4, 6, 8) alloys as a function of temperature. The present theoretical results show that the GSFE can be tuned by adding Al or changing the temperature. In particular, the intrinsic and extrinsic stacking fault energies increase, whereas the unstable stacking and twinning fault energies decrease with increasing temperature and Al doping. The thermodynamic phase stability can reflect *γ*_isf_ accurately due to the fact that *γ*_0_ is very close to *γ*_isf_. The interfacial energy *σ* slightly decreases with increasing temperature and Al content, but the change within the present composition-temperature interval always remains below ~30% compared to its mean value. Furthermore, from two parameters for twinning ability, it is predicted that Al addition and temperature increase cause a small decrease of the ability of twinning, but the alloys still remain prone to twinning even at the largest temperature and the highest Al-level considered here. The present theoretical data is expected to serve as input for modeling and design of new HEAs with desired mechanical properties.
